# Phase Morphology and Mechanical Properties of Cyclic Butylene Terephthalate Oligomer-Containing Rubbers: Effect of Mixing Temperature

**DOI:** 10.3390/ma9090722

**Published:** 2016-08-24

**Authors:** István Zoltán Halász, Tamás Bárány

**Affiliations:** 1 Department of Polymer Engineering, Faculty of Mechanical Engineering, Budapest University of Technology and Economics, Műegyetem rkp. 3, H-1111 Budapest, Hungary; halaszi@pt.bme.hu; 2 MTA-BME Research Group for Composite Science and Technology, Műegyetem rkp. 3, H-1111 Budapest, Hungary

**Keywords:** styrene-butadiene rubber, nitrile rubber, cyclic butylene terephthalate oligomer, CBT, bifunctional additive, semi-active filler plasticizer, processing aid, compounding temperature

## Abstract

In this work, the effect of mixing temperature (T_mix_) on the mechanical, rheological, and morphological properties of rubber/cyclic butylene terephthalate (CBT) oligomer compounds was studied. Apolar (styrene butadiene rubber, SBR) and polar (acrylonitrile butadiene rubber, NBR) rubbers were modified by CBT (20 phr) for reinforcement and viscosity reduction. The mechanical properties were determined in tensile, tear, and dynamical mechanical analysis (DMTA) tests. The CBT-caused viscosity changes were assessed by parallel-plate rheometry. The morphology was studied by scanning electron microscopy (SEM). CBT became better dispersed in the rubber matrices with elevated mixing temperatures (at which CBT was in partially molten state), which resulted in improved tensile properties. With increasing mixing temperature the size of the CBT particles in the compounds decreased significantly, from few hundred microns to 5–10 microns. Compounding at temperatures above 120 °C and 140 °C for NBR and SBR, respectively, yielded reduced tensile mechanical properties most likely due to the degradation of the base rubber. The viscosity reduction by CBT was more pronounced in mixes with coarser CBT dispersions prepared at lower mixing temperatures.

## 1. Introduction

Owing to their high elasticity and damping capability, rubber materials are widely used in industry and everyday life besides other polymers. However, conventional chemically crosslinked rubbers have some disadvantages, especially due to the complexity of compounds and processing. Numerous base rubbers and additives are required to fit the properties of the material to the specification of the final product. Mechanical and wear properties of cured rubbers are generally very poor. This note holds especially for synthetic rubber-based compounds. Thus various fillers (mostly carbon black, and inorganic mineral fillers) are used in the compounds as reinforcing agents. However, these fillers usually have drawbacks from the point of processing, because they cause a significant increment in the viscosity of the rubber raw mixtures, thus hindering their processability. This effect can be compensated by plasticizers and processing aids, but usually at the cost of the mechanical properties. This opposing effect could be resolved by an additive which could lower the viscosity of the raw mixtures and act as a reinforcing agent in the vulcanized rubber.

Oligomers are macromolecules that consist of few repeating units, contrary to polymers with hundreds, or thousands, of repeating units. Cyclic butylene terephthalate (CBT), as a macrocyclic oligomer, is one of these substances. It can be found in conventional polybutylene terephthalate (PBT) produced by polycondensation at levels of 0.25–2 wt % and can be produced in larger scales for example via cyclo-depolymerization of conventional PBT [1,2]. Due to its relatively low molecular weight it has a water-like melt viscosity (about 0.03 Pas at 190 °C) and the ability to polymerize into PBT (usually denoted as pCBT in the literature) in the presence of a suitable catalyst. The catalyst has a great effect on the conversion kinetics and the time required for the polymerization, thus these properties can be tailored to various processing techniques (i.e., reactive injection molding, or resin transfer molding) [3]. Polymerization time is also highly influenced by temperature, and since crystallization and melting enthalpies are closely matched the whole process seems to be athermic. This is also beneficial from the perspective of processing [4]. These properties make it as a promising matrix material for various nano- [5,6,7] and microcomposites [8,9] and carrier of different masterbatches. Without catalysts the CBT oligomer can be used as a viscosity reducer for many thermoplastic resins [10]. 

Previous studies showed that in rubber mixtures both polymerized, partly-polymerized [11,12,13], and unpolymerized [14] CBT may have a reinforcing effect. In peroxide cured hydrogenated acrylonitrile butadiene rubber (HNBR) CBT formed plate-like crystals during cooling, and had a notable reinforcing effect. CBT conversion to polymerized CBT (pCBT) was found relatively low (ca. 11% after curing 25 min at 190 °C), however, with a subsequent annealing at 250 °C for 3 h a conversion rate of ca. 70%–90% could be achieved [11]. Wear properties of the corresponding compounds were also investigated. Both CBT and pCBT caused a remarkable decrease in the coefficient of friction and specific wear rate of the tested compounds. Our previous work [14] showed that the viscosity of various rubber raw mixtures can be significantly decreased by the introduction of CBT in the rubber compounds. Moreover, the above-mentioned reinforcing effect was also present in synthetic rubber compounds, and this reinforcing effect increased with increasing polarity of the base rubber.

The focus of this present study is to investigate the effect of the CBT-rubber mixing temperature (below and above the melting range of CBT) on the mechanical and morphological properties of the tested rubbers. In order to investigate the effect of base rubber polarity both polar (acrylonitrile butadiene rubber, NBR), and apolar (styrene butadiene rubber, SBR) rubbers were involved in this study.

## 2. Materials and Methods

### 2.1. Materials and Processing

Base rubbers are listed in Table 1.

The oligomer was CBT 100^®^ of Cyclics Europe GmbH (Schwarzheide, Germany). The other ingredients used were: zinc-oxide (ZnO 500, provided by Zinc Oxide LLC, Dickson, TN, USA), stearic acid (Radiacid 0444, product of Oleon, Ertvelde, Belgium). 2,2-dibenzothiazole disulfide (MBTS) and sulphur were purchased from Ningbo Actmix Polymer (Ningbo, China) under the trade names of Curekind^®^MBTS and Curekind^®^Sulphur, respectively. The formulation of the rubber mixtures was the following: base rubber 100 part, CBT 20 part per hundred rubber (phr), ZnO 3 phr, stearic acid 2 phr, MBTS 1.5 phr, sulphur 2 phr. 

Mixes were made on a laboratory two-roll mill (Labtech LRM-SC-110, Labtech Engineering Co. Ltd., Samutprakarn, Thailand) at temperatures of: 40, 70, 100, 120, 140, 160, and 170 °C with the friction set to 1.3 for the CBT, and at 70 °C with a friction of 1.3 to curatives and other additives. The rubber mixes were cured at 170 °C under 2 MPa pressure in a Collin Teach-Line Platen Press 200E laboratory press (Dr. Collin GmbH, Ebersberg, Germany). Curing times (t_0.9_-time corresponding to 90% curing) were obtained from the curing curves (for curing data see Table 2). Specimens for further testing were cut from the produced sheets with a thickness of ca. 2 mm. 

### 2.2. Testing Methods

Curing properties and absolute values of the complex viscosities of the raw mixtures (i.e., without curatives) were studied a TA AR 2000 parallel plate rheometer (TA Instruments, New Castle, DE, USA), at 170 °C with a sinusoidal oscillation. The strain amplitude and oscillation frequency were 1% and 10 rad/s for curing tests and 25% and 40 rad/s for viscosity measurements. Curing curves were recorded in a time scale of 30 min, viscosity measurements were 3 min. The gap was set to 1.5 mm between the parallel plates.

Melting of CBT was monitored by DSC technique on a TA Q2000 DSC machine in N_2_ atmosphere with one heating. The heating rate was 10 °C/min in 0–250 °C range.

Tensile and tear tests were made on a Zwick Z250 universal testing machine equipped with a 20 kN load cell (Zwick GmbH, Ulm, Germany). For tensile tests, Type 1 specimens of DIN 53504 standard with a clamping length of 60 mm were loaded at 500 mm/min crosshead speed. Tear tests were made with the same test speed by the ASTM D624 standard (Type C specimen), with a clamping length of 56 mm. Both tests were run at room temperature.

Hardness tests were made by a Zwick H04.3150 hardness tester (Zwick GmbH, Ulm, Germany), by DIN 53505 standard with Shore A head using 12.5 N load.

Dynamic mechanical properties of the compounds were observed using a TA Q800 DMTA machine (TA Instruments, New Castle, DE, USA) in tensile mode on rectangle specimens with dimensions of ca. 2 × 2.5 × 10 mm^3^ (thickness × width × clamped length). Tests were run in the range of −100–100 °C with 3 °C/min heating rate at 10 Hz frequency with 0.01 N preload, and superimposed 0.01% sinusoidal strain. 

Apparent crosslink densities were calculated using the plateau moduli of the compounds. According to the rubber elasticity theory, the inverse of the plateau modulus (*E*_pl_) at temperatures above the glass transition temperature (T_g_) correlates with the mean molecular mass between the crosslinks (*M*_c_, (g/mol):

(1) $$M_{c}=\frac{3\rho \text{RT}}{E{’}_{\text{pl}}},$$

where ρ is the density [kg/m^3^], R is the universal gas constant [8.314 J/Kmol], T is the absolute temperature [K], (T = 293 K) *E*_pl_ is the plateau modulus [Pa].

The apparent crosslink density:

(2) $$\nu_{c}=\frac{\rho }{M_{c}}\;=\frac{E{’}_{\text{pl}}}{3RT},$$

where *ν*_c_ is the apparent crosslink density [mol/m^3^].

In order to gain insight into the morphology of the related compounds, SEM images were taken from the fracture surfaces of the tensile specimens. Cut surfaces were also investigated, at which specimens were cut by a razor blade in a pre-stretched state. SEM images were taken using a Jeol JSM-6380LA (Jeol Ltd., Tokyo, Japan). Prior to investigation, fracture surfaces were sputter-coated with gold. The images were captured using the secondary electron detector of the microscope, the accelerating voltage was 15 kV.

## 3. Results

### 3.1. Melting of CBT

**Figure 1:**
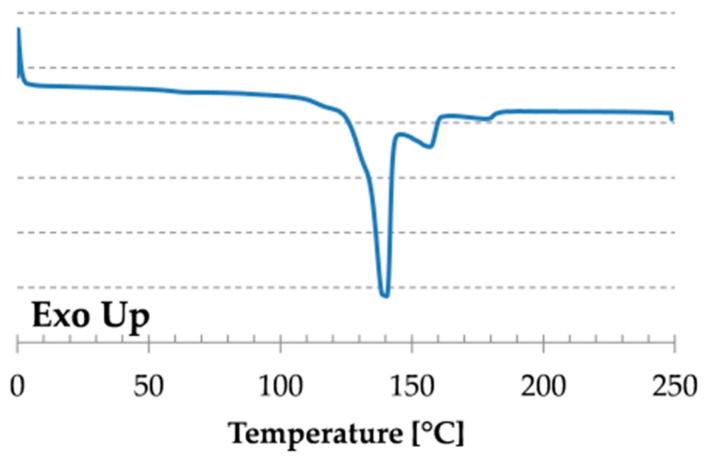
DSC curve of neat CBT.

Figure 1 shows the melting behavior of CBT based on its DSC curve. One can see that there are three well-defined peaks with peak temperatures of 140.4, 156.7, and 178.2 °C and a slight shoulder around 115 °C can also be observed. These are related to the melting of the rings with different sizes. Based on this data melting of CBT seems to occur in the temperature range of ca. 100–185 °C, but most of the CBT melts until ca. 150 °C.

### 3.2. Rheological Properties

**Figure 2:**
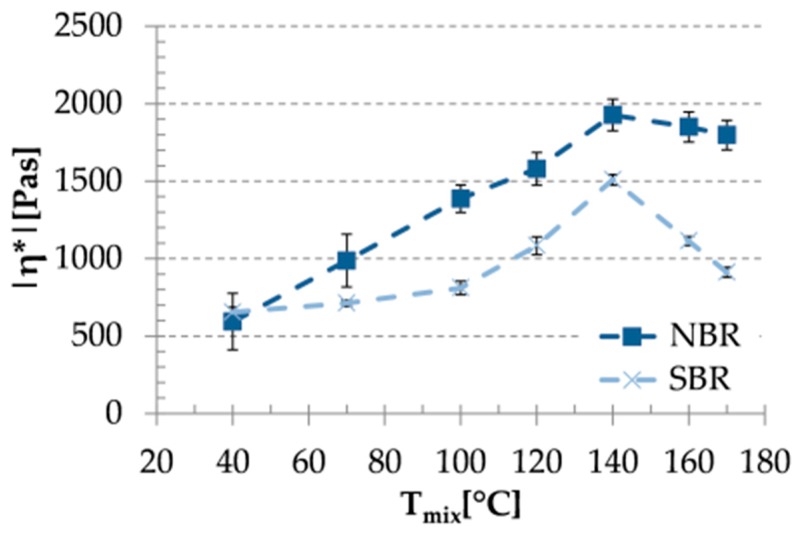
Absolute values of the complex viscosities of the uncured mixes as a function of mixing temperature.

Results of the viscosity measurements are depicted in Figure 2. At lower mixing temperatures (T_mix_) increasing T_mix_ resulted in increasing viscosities for both NBR- and SBR-based compounds. The viscosity of the mix produced at T_mix_ = 140 °C showed maximum beyond which the viscosity of the related mixes decreased.

### 3.3. Curing Properties

As curing curves, the shear storage moduli of each compound were recorded as a function of time. Based on these curves, minimal and maximal storage moduli were determined and listed in Table 2. One can see that in both rubber systems a significant drop can be seen at higher temperatures (for NBR at 170 °C, for SBR at 160 and 170 °C, respectively) and the G’_max_ values. Curing times (t_0.9_) were not strongly affected by mixing temperatures, except for at the highest T_mix_ (170 °C). In this case, the curing process was significantly slower, the curve itself showed a retarded, elongated curing. 

### 3.4. Mechanical Properties

**Figure 3:**
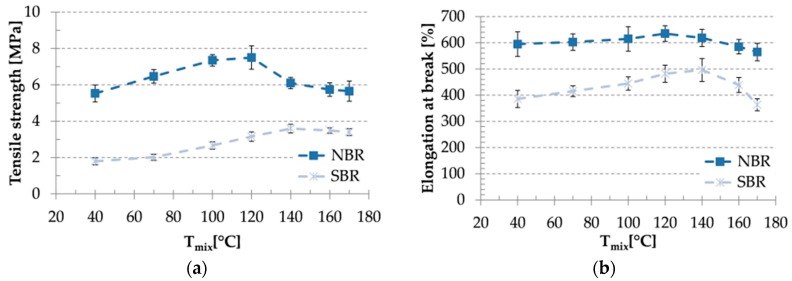
Result of tensile tests: (**a**) tensile strength and (**b**) elongation at break values as a function of mixing temperature.

Tensile results are shown in Figure 3. It can be clearly seen, that tensile strength and elongation at break values depend strongly on T_mix_. In temperature range of 40 and 120 °C for NBR and 40 and 140 °C for SBR an increasing tendency (just very slightly for elongation at break values for NBR based compounds) can be observed which suggests the increasing reinforcing effect of CBT. The tensile strength of NBR based compounds above T_mix_ of 120 °C and elongation at break values of SBR mixtures above 140 °C showed a remarkable reduction. Within the investigated T_mix_ range, highest tensile strength and elongation at break values were detected for mixes produced at 120 °C for NBR-based and at 140 °C for SBR-based systems. 

**Figure 4:**
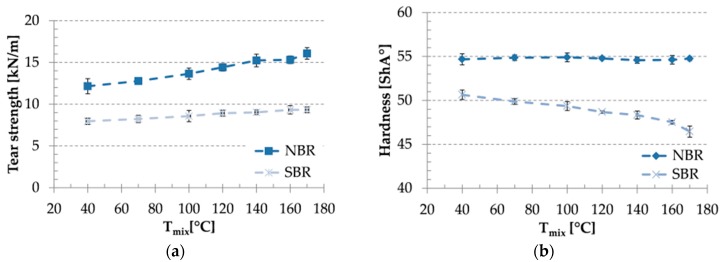
Tear strength (**a**) and hardness (**b**) values as a function of mixing temperature.

Tear strength values of the tested compounds (Figure 4) increased as a function of T_mix_. This effect is more pronounced for NBR-based mixes, than for SBR-based ones. Hardness values are also depicted in Figure 4. Results show that T_mix_ does not affect the hardness of NBR based compounds. SBR based compounds showed decreasing hardness with increasing T_mix_.

Data derived from DMTA measurements are listed in and Table 3.

In NBR based mixes, a marginal increase can be noted for the glass transition temperature, plateau modulus, and apparent crosslink density at T_mix_ of 170 °C. Similar effects are observable for the SBR-based compounds, but more pronouncedly for plateau moduli and apparent crosslink density of the compounds with T_mix_ of 120 °C and above. In the latter case, however, a noticeable decrease in the maximum values of tanδ was found. 

### 3.5. Morphology

**Figure 5:**
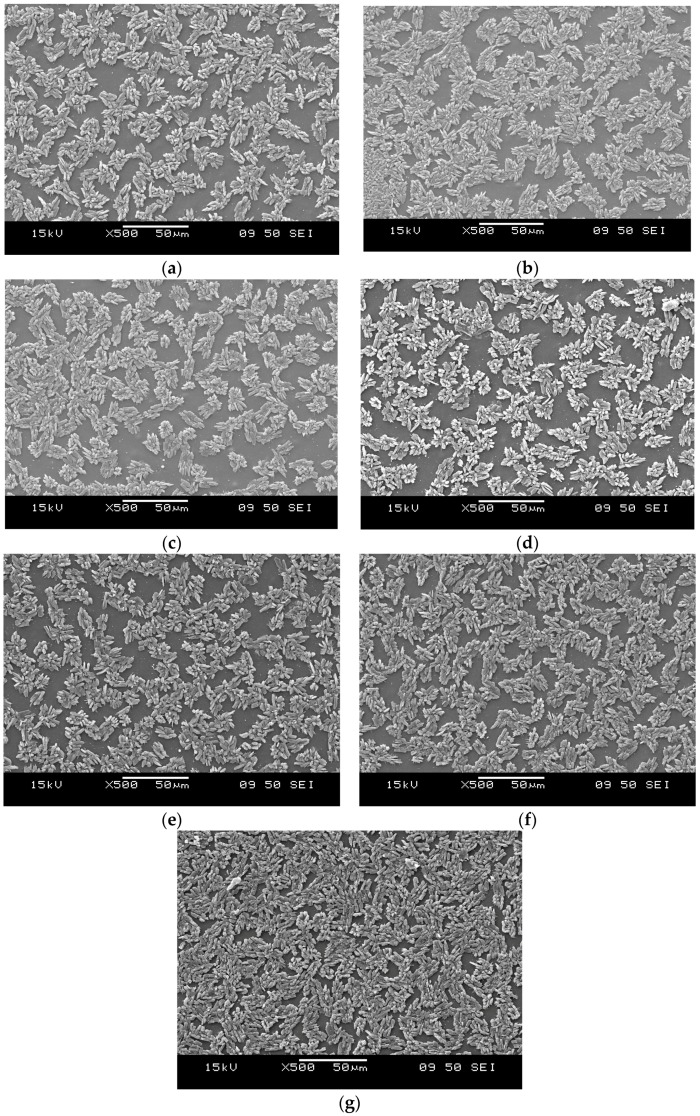
Fracture surfaces of the NBR-based rubbers mixed at (**a**) 40; (**b**) 70; (**c**) 100; (**d**) 120; (**e**) 140; (**f**) 160; and (**g**) 170 °C.

**Figure 6:**
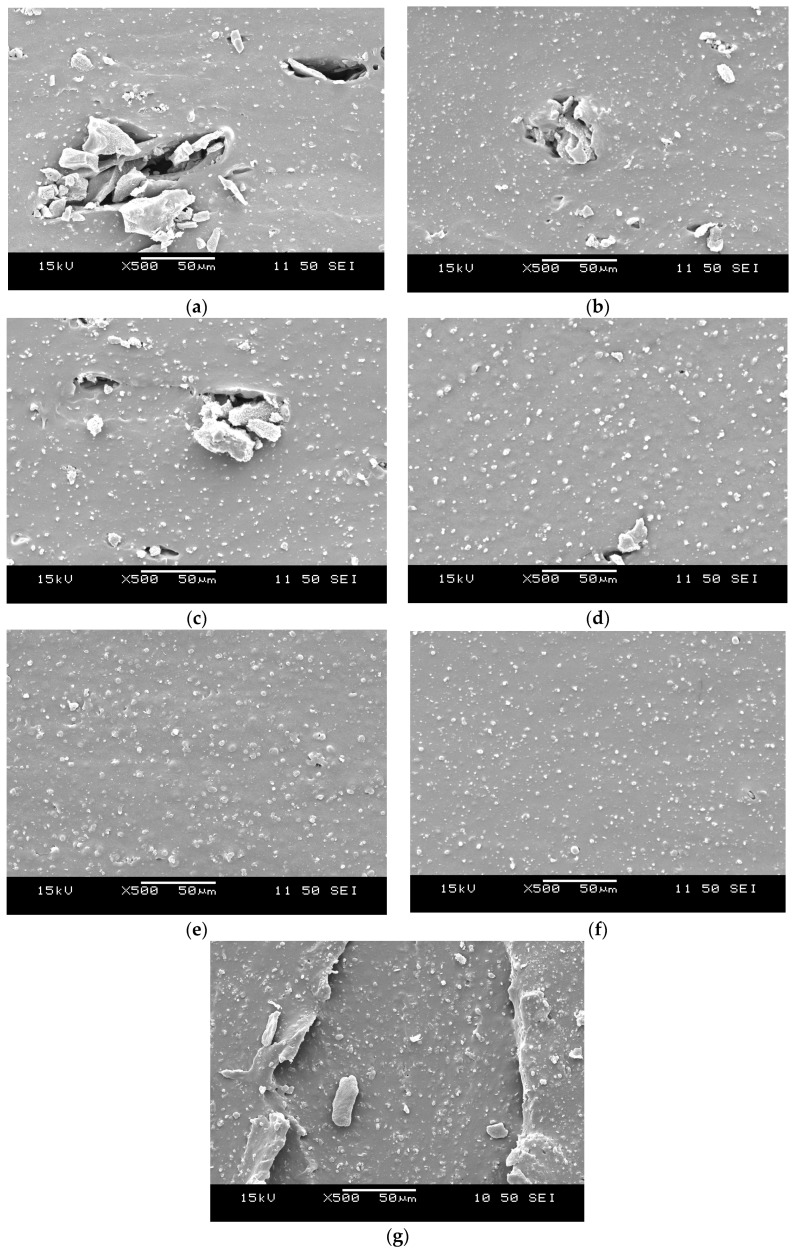
Fracture surfaces of the SBR rubbers mixed at (**a**) 40 °C; (**b**) 70 °C; (**c**) 100 °C; (**d**) 120 °C; (**e**) 140 °C; (**f**) 160 °C; and (**g**) 170 °C.

Figure 5 and Figure 6 show the SEM images of the fracture surfaces of tensile specimens of NBR- and SBR-based rubbers, respectively. CBT recrystallized during cooling in both rubbers and formed a well-defined secondary phase, but with fairly different appearance. Investigating the fracture surfaces of NBR based mixes (Figure 5) it can be clearly concluded that mixing temperature does not influence the phase morphology, so the final morphology seems to form during the curing process of the rubbers. In all mixes prism- and plate like crystals are present on the surface with the average size of few microns. This shows good agreement with data reported in literature for the appearance of CBT phase in rubbers [11,12,13,14]. In SBR based mixes (Figure 6) T_mix_ has definitely a strong impact on the morphology of CBT phase. 50–100 µm sized agglomerates can be seen in the T_mix_ range of 40–100 °C. At 120, 140, 160 and 170 °C these bulky cracked agglomerates are replaced by finer lamellar-like structures. In rubbers mixed at 170 °C the fracture surface become harsher, compared to lower temperature compounds. 

**Figure 7:**
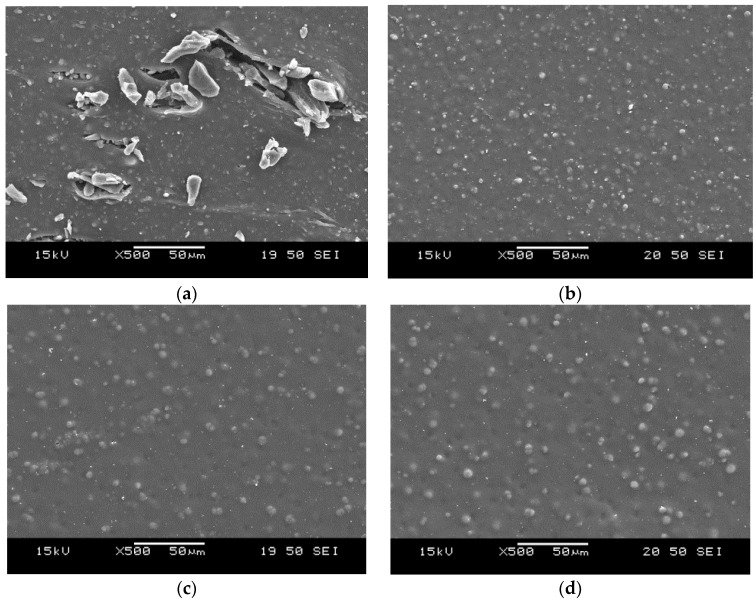
Cut surfaces of the SBR rubbers mixed at (**a**) 40 and (**b**) 140 and NBR rubbers mixed at (**c**) 40 and (**d**) 120 °C.

SEM pictures taken from cut surfaces of the rubbers are shown in Figure 7. Based on these pictures, it can also be stated that—in SBR mixes—the phase morphology depends far more on the mixing temperature than in NBR-based mixtures. At lower mixing temperatures, where melting of CBT does not occur during mixing, a coarse structure formed (similar as seen on the fracture surfaces). In case of cut surfaces of NBR-based mixtures, a far more different structure can be seen compared to the related fracture surfaces.

Similar to SBR mixtures at higher mixing temperatures (120 °C and above) CBT phase is present in spherical-like structures with a diameter of around 5–10 μm. This structure seems independent from mixing temperature. Very similar structure could be observed in all NBR mixtures regardless to their mixing temperatures. The other interesting finding is the difference between fracture and cut surfaces of NBR mixtures. Both seem to be independent of the mixing temperature, but while in the former plate-like and prismatic CBT crystals are present, the latter contains a CBT phase with a spherical structure.

## 4. Discussion

Based on our present result, it can be stated that elevating the mixing temperature of CBT into rubbers is an effective and promising way to enhance their mechanical properties. At lower temperatures (below the melting temperature of the oligomer) in apolar rubbers, such as SBR, CBT forms a coarse structure with easily cracking bulky agglomerates with a size up to few hundred microns. In polar rubbers, such as NBR, the morphology does not change as a function of the mixing temperature, so the final morphology seems to form at the curing process of the rubber. However, the reinforcing effect of CBT increases with increasing temperatures (up to 120 °C in our work). This can be explained by improving interphase bonding of the CBT and the rubber matrix. Both SBR- and NBR-based compounds had an “optimum” mixing temperature above which the mechanical properties deteriorated. This can most likely be traced to the degradation of the base rubber. This statement is in line with changes observed for the viscosity and tensile mechanical values, and even for curing curves. Considering the latter it can be seen, that in the case of higher mixing temperature (160, 170 °C for NBR, and 140, 160, and 170 °C for SBR) the final moduli of the mixtures start to decrease. In NBR-based rubbers, during failure of the material the shape of the CBT phase agglomerates undergo a dramatic alteration. “Ball-like” spherical structures seem to break up or pop-out to form a quasi-continuous layer on the fracture surface. Here, CBT crystals seem to form crystal-clusters with plate-like and prismatic crystals. This suggests that during the failure of NBR-based compounds crack propagation seems to occur through the particles of the CBT phase. This leads to a fracture surface on which CBT is over represented compared to its weight fraction in the compound. DMTA results suggests, that the final crosslink densities of the compounds with T_mix_ of 170 °C for NBR and 140, 160, and 170 °C for SBR are higher, than at lower mixing temperatures. Decreasing maximum values of tanδ and increasing plateau moduli suggests hindered molecular segment mobility, which might be caused by increasing crosslink density meaning shorter molecular segments between crosslinks. 

## 5. Conclusions

Based on our work the following conclusions can be drawn:

In SBR-based mixtures increasing mixing temperatures led to finer dispersion of the CBT phase. Finer dispersion of CBT agglomerates caused an improvement in mechanical properties.

In NBR based mixtures, the phase morphology proved to be practically independent of the mixing temperature. It is assumed that the final structure is formed in the curing step. Regardless to the unchanged morphology, higher mixing temperatures (up to 120 °C) yielded improvements in mechanical properties of the NBR mixes. In NBR-based compounds, the CBT phase underwent a remarkable change during failure of the rubber. Cut surfaces showed spherical CBT agglomerates, while on the fracture surfaces a quasi-continuous “CBT layer” could be observed with prismatic and plate-like crystals. 

## Figures and Tables

**Table 1:** Types and producers of rubbers used.

Abbreviation	Producer, Type	Main Properties
NBR	Lanxess, Perbunan^®^ 3945F	Mooney viscosity (ML, 1 + 4, 100 °C): 45 ± 5
SBR	Goodyear Chemical, Plioflex^®^ 1502	Mooney viscosity (ML, 1 + 4, 100 °C): 44 Bound styrene content: 23.5 m%

**Table 2:** Curing data derived from curing curves.

Rubber	Mixing Temperature [°C]	G’_min_ [kPa]	G’_max_ [kPa]	t_0.9_ [min]
NBR	40	13.1	694.9	14.5
	70	17.7	753.8	13.4
	100	23.4	759.4	13.6
	120	20.2	770.7	13.5
	140	26.8	897.2	13.5
	160	19.5	827.4	13.6
	170	20.9	762.9	14.3
SBR	40	40.1	399.0	13.3
	70	44.9	335.8	14.6
	100	44.2	409.2	14.7
	120	40.7	400.0	14.4
	140	28.9	308.3	14.6
	160	5.4	175.5	12.4
	170	5.3	147.9	22.4

**Table 3:** Storage moduli, apparent crosslink densities, tanδ values, and glass transition temperatures of the tested compounds.

Rubber	Mixing Temperature [°C]	E’_pl_ [MPa]	ν_c_ [mol/m^3^]	tanδ_max_ [-]	T_g_ [°C]
NBR	40	8.31	1137.0	1.40	1.2
	70	8.22	1124.7	1.41	1.1
	100	8.77	1200.0	1.42	1.4
	120	8.20	1122.0	1.42	1.7
	140	8.21	1123.4	1.44	1.4
	160	8.64	1182.2	1.41	2.3
	170	9.35	1279.3	1.40	3.4
SBR	40	5.95	814.1	1.17	−27.9
	70	6.09	833.3	1.21	−28.3
	100	5.79	792.2	1.20	−28.4
	120	6.39	874.3	1.19	−27.8
	140	6.92	946.9	1.14	−27.0
	160	7.21	986.5	1.08	−24.9
	170	8.41	1150.7	0.87	−23.3
